# Downregulation of PAX6 by shRNA Inhibits Proliferation and Cell Cycle Progression of Human Non-Small Cell Lung Cancer Cell Lines

**DOI:** 10.1371/journal.pone.0085738

**Published:** 2014-01-15

**Authors:** Xiaoting Zhao, Wentao Yue, Lina Zhang, Li Ma, Wenyun Jia, Zhe Qian, Chunyan Zhang, Yue Wang

**Affiliations:** Department of Cellular Biology, Beijing TB and Thoracic Tumor Research Institute/Beijing Chest Hospital, Capital Medical University, Beijing, China; Ottawa Hospital Research Institute, Canada

## Abstract

**Background:**

The transcription factor PAX6 is primarily expressed in embryos. PAX6 is also expressed in several tumors and plays an oncogenic role. However, little is known about the role of PAX6 in lung cancer.

**Methods:**

The function of PAX6 in lung cancer cells was evaluated by small interfering RNA-mediated depletion of the protein followed by analyses of cell proliferation, anchorage-independent growth, and cell cycle arrest. The changes of cyclin D1, pRB, ERK1/2, p38 expression caused by PAX6 inhibition were detected using western-blotting. The PAX6 mRNA level in 52 pairs of tumors and corresponding matched adjacent normal tissues from non-small cell lung cancer patients and lung cancer cell lines was detected by real-time PCR.

**Results:**

Suppression of PAX6 expression inhibited cell growth and colony formation in A549 and H1299 cells. The percentage of cells in G1-phase increased when PAX6 expression was inhibited. The cyclin D1 protein level, as well as the pRB phosphorylation level, decreased as a result of PAX6 down-regulation. The activity of ERK1/2 and p38 was also suppressed in PAX6 knock-down cells. The PAX6 mRNA was highly expressed in lung cancer tissue and lung cancer cell lines. In most patients (about 65%), the relative ratio of PAX6 mRNA in primary NSCLC versus adjacent tissues exceeded 100.

**Conclusions:**

Our data implicated that PAX6 accelerates cell cycle progression by activating MAPK signal pathway. PAX6 mRNA levels were significantly elevated in primary lung cancer tissues compared to their matched adjacent tissues.

## Introduction

A recent overview on global cancer statistics showed that lung cancer was the most commonly diagnosed cancer, as well as the leading cause of cancer death [1]. Early detection and targeted therapy is a potential method for lung cancer prevention and therapy [2]. It is important to find which pathways or proteins are active in lung tumor progression [3]. On the basis of the "cancer stem cell hypothesis," tumors are thought to originate through tissue-specific stem cell expression [4]–[6]; in other words, tumors are attributed to stem cell factor overexpression [3], [5], [7]. Paired-box 6 (Pax6) is an important transcription factor during embryogenesis and a stem cell factor [3]. Hence, PAX6 may play an important role in tumorigenesis.

PAX6 belongs to the PAX gene family, which encodes a group of nine paired-box transcription factors with important roles in development and disease [3]. PAX6 is an important transcription factor in development of the eyes, pancreas, and central nervous system [3], [8]. PAX6 expression was recently found in tumors, suggesting an oncogenic role [9]. PAX6 is frequently expressed in retinoblastoma, pancreatic tumors, and intestinal tumors [6], [10], [11]. PAX6 is also highly expressed in brain and breast cancer cell lines [9]. In pancreatic carcinoma cell lines, the inhibition of PAX6 expression leads to a decrease in cell growth and survival [12]. PAX6 is also a regulator of MET tyrosine kinase receptor expression in pancreatic carcinoma cell lines [12]. MET is a potential biomarker and therapeutic target for tumors, which confirms the oncogenic role of PAX6 in tumorigenesis [13].

It was previously reported that PAX8 and PAX5 are highly expressed in non-small cell lung cancer (NSCLC) and small cell lung cancer cell lines, respectively [14]; but little is known regarding PAX6 expression and function in lung cancer. In this study, we investigated whether PAX6 regulated cell proliferation of NSCLC. Our findings show that PAX6 promotes G1-S progression by activating the MAPK signal pathway. PAX6 mRNA was frequently expressed in lung cancer tissue as compared to corresponding adjacent non-neoplastic tissue. This suggests that PAX6 is a new potential target in lung cancer.

## Materials and Methods

RPMI 1640, fetal bovine serum (FBS), and Trizol Reagent were purchased from Invitrogen (Carlsbad, CA); M-MLV reverse transcription, CellTiter 96® aqueous non-radioactive cell proliferation assay, oligo-dT, and dNTP were obtained from Promega (Madison, WI); SYBR® Green PCR Master Mixture was from Applied Biosystems (Carlsbad, CA); anti-PAX6 antibodies were purchased from Abnova (Taibei, Taiwan), anti-pRB, -ERK1/2, p38, -pERK, -pp38, -cyclin D1, and -pRB (S780 phosphorylation) antibodies were obtained from Abcam (Cambridge, England, UK); and enhanced chemiluminescence (ECL) reagent was obtained from Pierce (Rockford, IL). Propidium iodide (PI), RNase A, and protease inhibitor cocktail were purchased from Sigma (St. Louis, MO).

### Samples

Fifty-two NSCLC specimens were obtained from patients undergoing surgical resection at Beijing Chest Hospital. Primary lung cancer samples and matched, adjacent normal tissues were used.

The study and use of specimens was reviewed and approved by Research Ethic Committee in Beijing Chest Hospital, Capital Medical University (Beijing, China). Written informed consent was obtained from all patients. The clinical characteristics of the patients are listed in Table 1.

**Table 1:** Patients and Clinical Characteristics.

Characteristics	Number of Patients
**Patient Age, Years**	
0 – 60	27
>60	25
**Gender**	
Male	40
Female	12
**Smoke Status**	
Nonsmoker	21
Smoker	31
**Histologic Type**	
SCC*	31
Adenocarcinoma	21
**Histological Grade**	
III	21
II	31
**Tumor Size**	
0 – 3 cm	14
>3 cm	38
**Lymph Node Status**	
Negative	27
Positive	25
**Distant Metastasis**	
Negative	44
Positive	8
**TNM Stage**	
Stage I	17
Stage II	10
Stage III	19
Stage IV	6

: Squamous Cell Carcinoma.

### Cell culture

Human lung adenocarcinoma cell lines A549 and NCI-H1299, human large cell lung carcinoma cell lines NCI-H460, small cell lung cancer cell line NCI-H446, human embryo lung fibroblasts (MRC-5) were obtained from the National Platform of Experimental Cell Resources Sci-Tech. Human large cell lung carcinoma cell lines, 95C, 95D and 801D were obtained from the tumor center of Chinese Academy of Medical Sciences. Human lung adenocarcinoma cell line A2 and squamous cell carcinoma cell line L were isolated and established by our lab. The lung cancer cell lines were cultured in RPMI 1640 medium (Invitrogen, Carlsbad, CA, USA) supplemented with 10% fetal bovine serum (FBS; Gibco, Los Angeles, CA, USA). MRC-5 were maintained in MEM-EBSS supplemented with 10% FBS.

### Construction of a PAX6 shRNA lentiviral vector and infection into cells

Four RNA interference (RNAi) candidate target sequences were designed based on the human *pax6* mRNA sequence and cloned into the pGCSIL-GFP vector (GeneChem, Shanghai, China). The RNAi sequence GAGTAGCGACTCCAGAAGT was the most effective at suppressing PAX6 mRNA in H1299 and A549 cells, and was used in subsequent experiments to knock down endogenous PAX6. Nonsilencing (NS)-small interfering RNA (shRNA) (TTCTCCGAACGTGTCACGT) was also cloned into the pGCSIL-GFP vector and used as a control (GeneChem). The recombinant virus was packaged in 293T cells using a Lentivector Expression System (GeneChem).

For cellular infection, H1299 and A549 cells were subcultured at 5,000 cells/well in 96-well culture plates and infected with lentivirus-mediated pax6-shRNA or NS-shRNA. The GFP expression level was detected via fluorescence microscopy (Nikon, Tokyo, Japan) to determine the infection efficiency.

### RNA isolation and real-time PCR

Total RNA from tissue and cells was isolated with Trizol Reagent according to the manufacturer’s protocol. The total RNA concentration was calculated by measuring the OD_260_ and the samples were stored at –80°C.

Total RNA (2 µg) was reverse-transcribed using an M-MLV Reverse Transcriptase Kit according to the manufacturer’s protocol. The cDNA (20 ng) was mixed with SYBR® Green Master Mix, and genes were amplified with appropriate primers using a real-time PCR detection system (ABI7500; Life Technologies, Carlsbad, CA). The relative expression levels of PAX6 mRNA were calculated by normalization to the β-actin mRNA level. The PCR primers used were as follows: PAX6 forward, 5'-TTCAGCACCAGTGTCTACCA-3'; PAX6 reverse, 5'-GCTGTAGGTGTTTGTGAGGG-3'; β-actin forward, 5'-TTAGTTGCGTTACACCCTTTC-3'; and β-actin reverse, 5'-GCTGTCACCTTCACCGTTC - 3'.

### Cell proliferation assay

A proliferation assay was carried out using Non-Radioactive Cell Proliferation Assay according to the manufacturer’s protocol. Briefly, 5,000 cells/well were seeded into 96-well culture plates in RPMI 1640 containing 10% FBS. The cells were cultured for 5 days, then 20 µL of 3-(4,5-dimethyl-thiazol-2yl)-5-(3-carboxymethoxyphenyl)-2- (4-sulfophenyl)-2H-tetrazolium (MTS) was added to each well and the cells were incubated at 37°C for 3 h every 24 h. The absorbance was recorded at 490 nm with a universal microplate reader (Bio-Rad, Hercules, CA). All the experiments were repeated three times. The data are presented as means ± SEM.

### Colony formation assay

Cells were seeded in triplicate at 300 cells/well in a 6-well plate. After 7 days of culture, the cells were washed twice with NaCl (0.9%), stained with 2% gentian violet for 20 min, washed with water, and air-dried. Foci were counted by microscopy. The experiments were repeated three times and data are presented as means ± SEM.

### Soft-agar assay

Cells (1,000) were seeded into 6-well plates in 2 mL of growth medium containing 0.3% agar and used to overlay 1.4-mL layers of growth medium containing 0.6% agar. After 21 days of culture, the colonies were counted. All the experiments were repeated three times. The data are presented as means ± SEM.

### Cell cycle analysis

Cells were harvested, washed with cold PBS twice and fixed in 70% ethanol at –20°C overnight. The cells were then centrifuged (1,500 rpm, 10 min) and washed twice using phosphate-buffered saline (PBS). Next, the cells were resuspended in 0.5 mL of PBS containing 50 µg/mL RNase A for 1 h at 37°C. The cells were then loaded with 65 μg/mL PI for 30 min in the dark at 4°C. The percentage of cells in different phases of the cell cycle was measured by flow cytometry (Beijing Determination of Traditional Chinese Medicine Research Institute). The experiments were repeated three times. The data are presented as means ± SEM.

### Western blotting

Cells were digested with trypsin and centrifuged. The cell pellet was washed twice with PBS. Next, the cells were disrupted in lysis buffer (10 mM Tris-HCl, pH 7.4, 1 mM EDTA, 0.1% Triton X-100, 0.1% SDS and 1× protease inhibitor cocktail) on ice for 15 min and centrifuged at 12,000 rpm for 20 min. Insoluble material was removed and protein concentrations were determined using a bicinchoninic acid kit. For Western blot analysis, cell lysates (30 μg/well) were subjected to SDS-PAGE and transferred to nitrocellulose filter membranes. The membranes were incubated with primary antibodies (anti-PAX6, -ERK1/2, p38, -pERK, -pp38, -cyclin D1, -RB, or -RB S780 phosphorylation) overnight at 4°C. Secondary antibodies conjugated with horseradish peroxidase were subsequently used. Signals were detected using ECL and exposed to Kodak X-OMAT film. The results were scanned and analyzed using Alpha View Analysis Tools.

### Statistical analysis

All values are expressed as the mean ± SEM. Through real-time RT-PCR, MTS assay, colony formation, soft-agar assays, cell cycle analysis, and western-blot assay, for comparison between means of 2 groups, statistical differences were tested with unpaired Student *t*-tests. Statistical significance was tested using SPSS Statistics, version 13.0. P<0.05 (*) was considered different; P<0.01 (**) was considered significantly different.

## Results

### PAX6 mRNA expression was inhibited in cells infected with the PAX6 shRNA lentiviral vector

**Figure 1:**
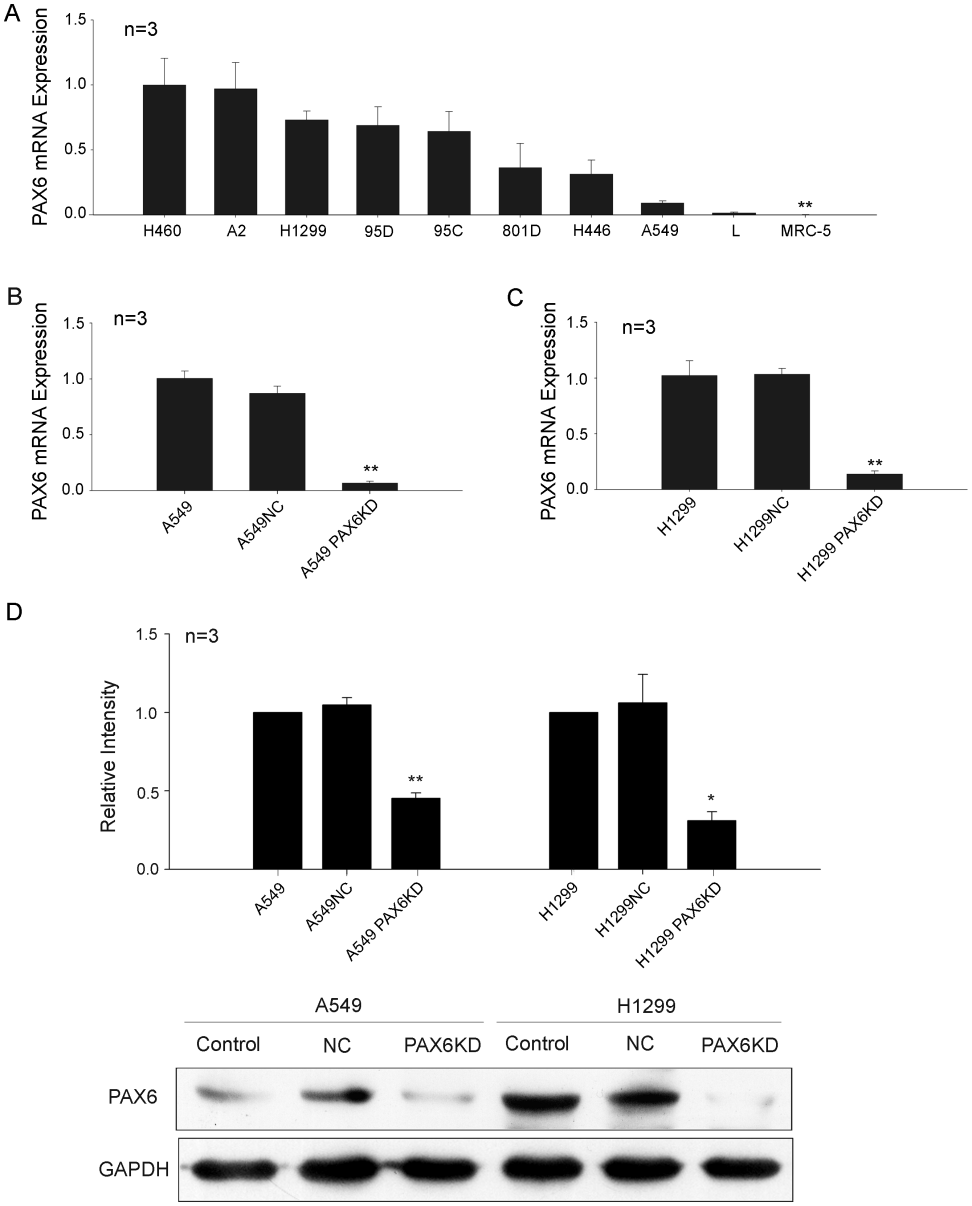
PAX6 mRNA was highly expressed in lung cancer cells and its expression was suppressed by pax6-shRNA. A, Real-time PCR analysis for the PAX6 mRNA expression level in H460, A2, 95C, 95D, H1299, H446, 801 D, A549, and L lung cancer lines, as well as in the normal human fetal lung fibroblast cell line MRC-5. B, -C, Confirmation of PAX6 mRNA knockdown by real-time RT-PCR assays performed on total RNA isolated from A549 (B) and H1299 (C) cells infected with pax6-shRNA, or a random shRNA. The PAX6 mRNA expression levels in A549 and H1299 cells were measured by quantitative real-time RT-PCR. The y-axis represents the normalized PAX6 mRNA expression relative to A549 (B) or H1299 (C) cells. **P < <0.01. D, The protein levels of PAX6 were determined by western-blot and GAPDH expression level was used as a control. Quantification was made by determining the gray level of PAX6 protein which was normalized against GAPDH levels. Data are expressed as mean ±SEM of independent experiments (times of the experiments are listed above the histograms). PAX6 expression was obviously weakened in A549 PAX6 KD and H1299 PAX6 KD cells.

PAX6 mRNA expression was determined in this study. As shown in Figure 1A, PAX6 was highly expressed in most lung cancer cell lines. In contrast, MRC-5, a normal human fetal lung fibroblast cell line, did not express PAX6 (Figure 1A).

To elucidate whether PAX6 expression has any effect on the growth of lung cancer cells, RNAi was used to generate pax6 knock-down (PAX6 KD) cell lines. We selected two target cell lines: H1299, which showed high levels of PAX6 expression, and A549, which showed low levels of expression. In the present study, pGCSIL-pax6 shRNA-GFP was infected into H1299 and A549 cells. Cells were also infected with pGCSIL-NS shRNA-GFP (PAX6 NS) as a negative control (NC). To determine the function of PAX6, H1299, H1299NC, A549, or A549NC cells were used as controls in all assays.

The PAX6 mRNA level in H1299 PAX6 KD and A549 PAX6 KD cells was determined by real-time PCR to confirm whether PAX6 expression was specifically inhibited through RNAi in A549 and H1299 cells. As shown in Figure 1B, PAX6 expression in A549 PAX6 KD cells was inhibited by 80–90% compared to cells infected with lentivirus-mediated NS-shRNA. We found similar results in H1299 PAX6 KD cells. PAX6 mRNA expression in these cells was also inhibited by 90–95% as compared to NC cells (**P<0.01; Figure 1C).

PAX6 protein expression in these cells was detected by Western blotting. As shown in Figure 1D, PAX6 protein in H1299 PAX6 KD and A549 PAX6 KD cells was not readily detected, whereas a clear PAX6 protein band was evident in the control cells.

### Inhibition of PAX6 expression leads to a decline in cell proliferation

**Figure 2:**
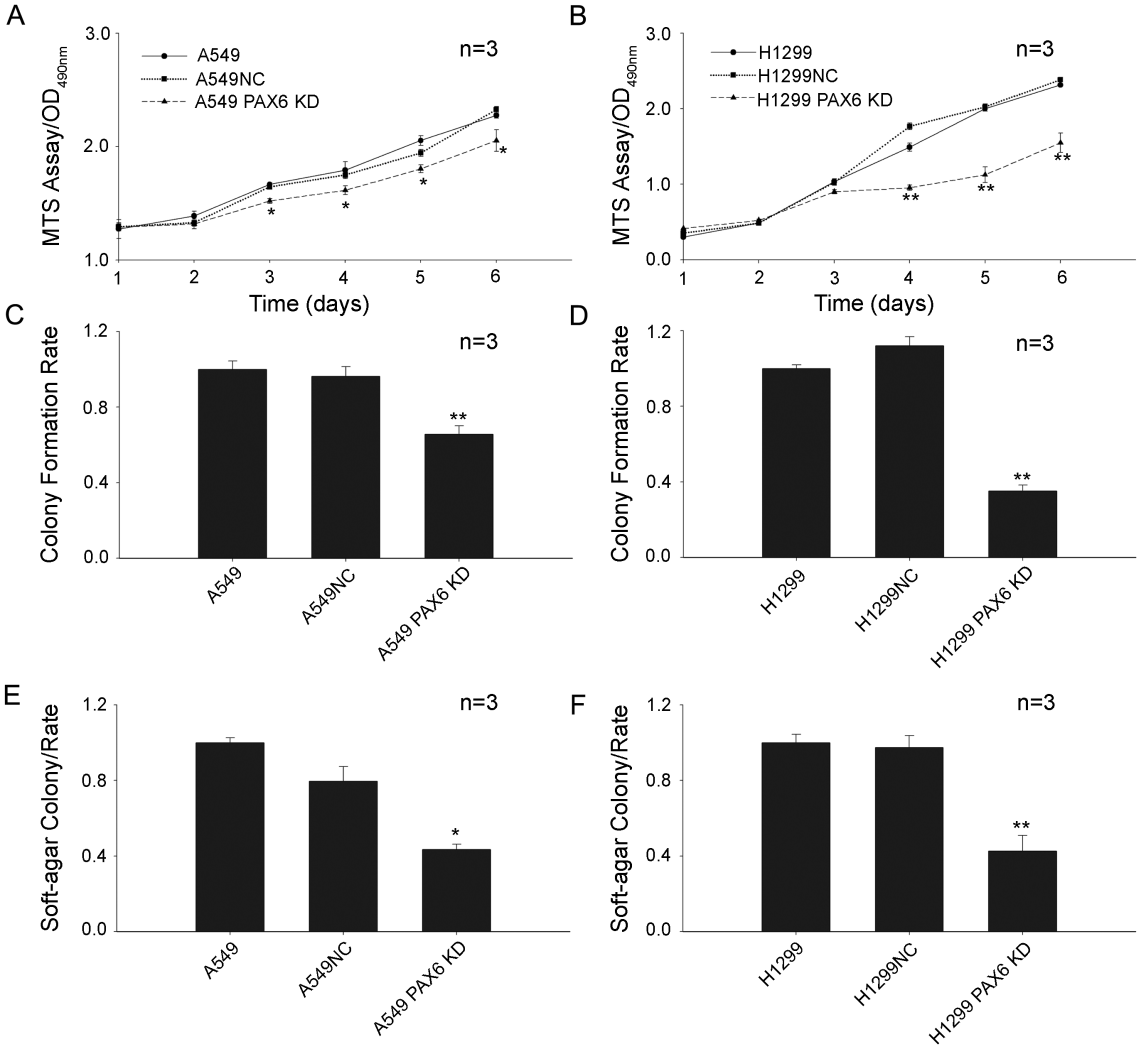
The lentivirus-mediated pax6-shRNA knockdown of PAX6 expression could suppress lung cancer cell growth. A, -B, A549 PAX6 KD, H1299 PAX6 KD cells, and control cells were seeded in 96-well plates and an MTS assay was performed. The absorbance at 490 nm (y -axis) was measured at 24-h intervals, up to 120 h. C, -D, Colony formation efficiency in A549 PAX6 KD, H1299 PAX6 KD cells, and control cells. The y -axis represents the normalized colony formation rate relative to A549 (C) or H1299 (D) cells. E, -F, A soft -agar assay was performed to investigate the effects of PAX6 on tumorigenesis in vitro. The y -axis represents the normalized soft -agar colony formation rate relative to A549 (E) or H1299 (F) cells. The data are expressed as the means ± SEM from three separate experiments. Times of the experiments are listed above the graph.*P <0.05, **P <0.01.

PAX6 is a critical transcription factor that plays an important role in regulating proliferation and differentiation during human embryonic development [3]. A cell proliferation assay was performed to determine whether PAX6 plays a role in cellular growth. A549 PAX6 KD, H1299 PAX6 KD, and control cells were seeded in 96-well plates and cell proliferation activity was measured using a Cell Proliferation Assay kit. A549 and H1299 cell growth was obviously suppressed when PAX6 expression was inhibited by RNAi (Figure 2A and B). As shown in Figure 2A and B, the decrease in cell growth caused by the inhibition of PAX6 expression in H1299 was much stronger than that in A549 cells. These different results may be attributable to the different PAX6 expression levels between H1299 and A549 cells displayed in Figure 1A and D.

### Reduced colony formation and soft-agar colony formation in PAX6 KD cells

Colony formation represents a loss of contact inhibition or the ability to maintain cell growth and movement despite contact with surrounding cells. To clarify whether PAX6 could confer a loss of contact inhibition, cells infected with pGCSIL-pax6 shRNA-GFP as well as their control cells were seeded into 6-well plates and cultured for 7 days. After 2% gentian violet staining, colonies containing more than 50 cells were counted under a light microscope. As displayed in Figure 2C and D, the inhibition of PAX6 expression in A549 and H1299 cells led to an obvious decrease in the number of foci generated compared to the control cells (**P<0.01).

To further study the function of PAX6, soft-agar colony formation was analyzed to determine whether PAX6 contributed to anchorage-independent colony formation in lung cancer cells. The rate of soft-agar colony formation declined in A549 PAX6 KD and H1299 PAX6 KD cells compared to NC cells (**P<0.01, *P<0.05; Figure 2E and F).

### PAX6 expression increased cell growth by promoting faster progression into S phase of the cell cycle

**Figure 3:**
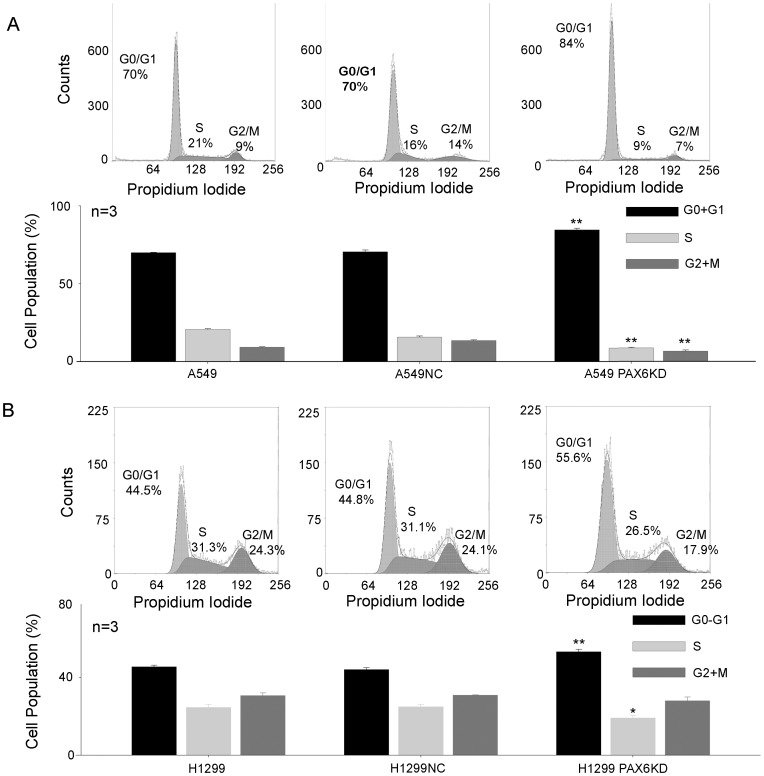
PAX6 expression promoted cell cycle progression. A, B, Cell cycle analysis. Cells were stained with propidium iodide (PI) and analyzed for cell cycle phase distribution. The histogram was the statistical data from three independent experimental replicates. *P <0.05, **P <0.01.

To detect the effect of PAX6 on cell cycle progression, the cell cycle progression of A549 PAX6 KD, H1299 PAX6 KD, A549 PAX6 NC, H1299 PAX6 NC, A549, and H1299 cells was analyzed by flow cytometry. As displayed in Figure 3A, the percentage of cells entering S phase was decreased in the A549 PAX6 KD cell line along with an increase in the population of G0-G1 phase cells. A similar result was observed in H1299 PAX6 KD, H1299 PAX6 NC, and H1299 cells (Figure 3B). In these experiments, PAX6 expression led to cell growth by inducing cell cycle progression.

**Figure 4:**
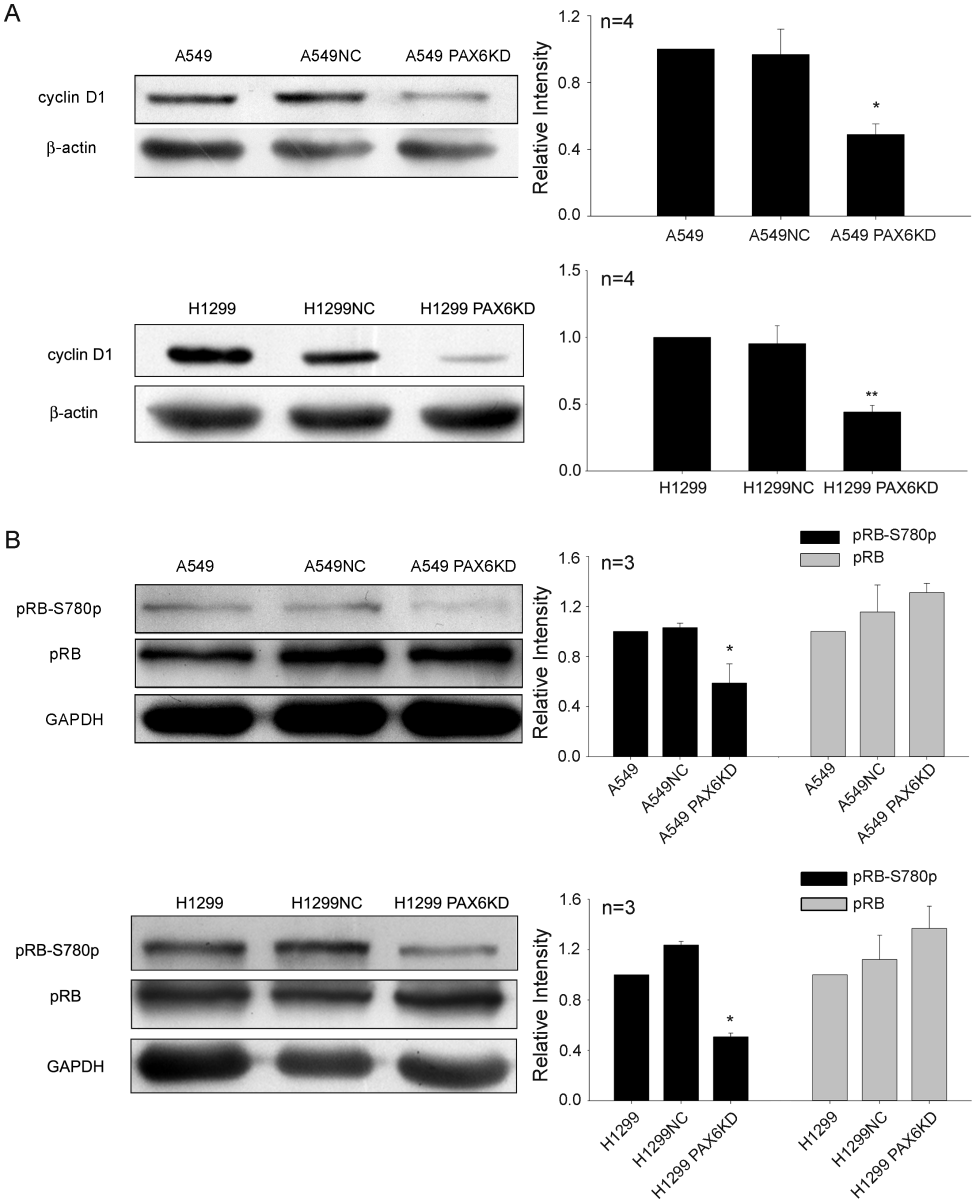
Cyclin D1 expression and pRB phosphorylation was inhibited while PAX6 expression was suppressed. A, B, The expression of cyclin D1, pRB and the phosphorylated pRB in A549 PAX6 KD, A549 NC, A549 cells as well as H1299 PAX6KD, H1299NC, H1299 cells was determined by Western blotting. β-actin and GAPDH expression level was measured as internal loading controls respectively. Cyclin D1 and pRB levels were measured by the gray level and were normalized by internal loading controls. Data are expressed as mean ±SEM. Times of the experiments are listed above the histograms. *P <0.05, **P <0.01.

In this study, the expression level of cyclin D1, a relevant cyclin regulating G1-S progression [15], [16], was detected in A549 PAX6 KD and H1299 PAX6 KD cells. As indicated in Figure 4A, cyclin D1 expression was decreased in A549 PAX6 KD cell lines compared to control cells. We found a similar result in H1299 PAX6 KD cells (Figure 4A). Another relevant cyclin regulating G1/S progression is cyclin E [17]. We also determined whether cyclin E was regulated by PAX6 expression. As a result, cyclin E expression was not affected by the stable shRNA-mediated knockdown of PAX6 in lung cancer cells (data not shown). This demonstrates that PAX6 might promote cell growth by inducing cyclin D1 expression.

The major substrate of cyclin D1-CDK4/6 complexes is retinoblastoma protein (pRB) [18]. Thus, pRB S780 protein phosphorylation was also detected by Western blotting (Figure 4B). The S780 phosphorylation of pRB was decreased when PAX6 expression was inhibited in A549 cells. A similar result was obtained when H1299 PAX6 KD cells were used (Figure 4B).

### MAPK signal pathway was suppressed by the inhibition of PAX6

**Figure 5:**
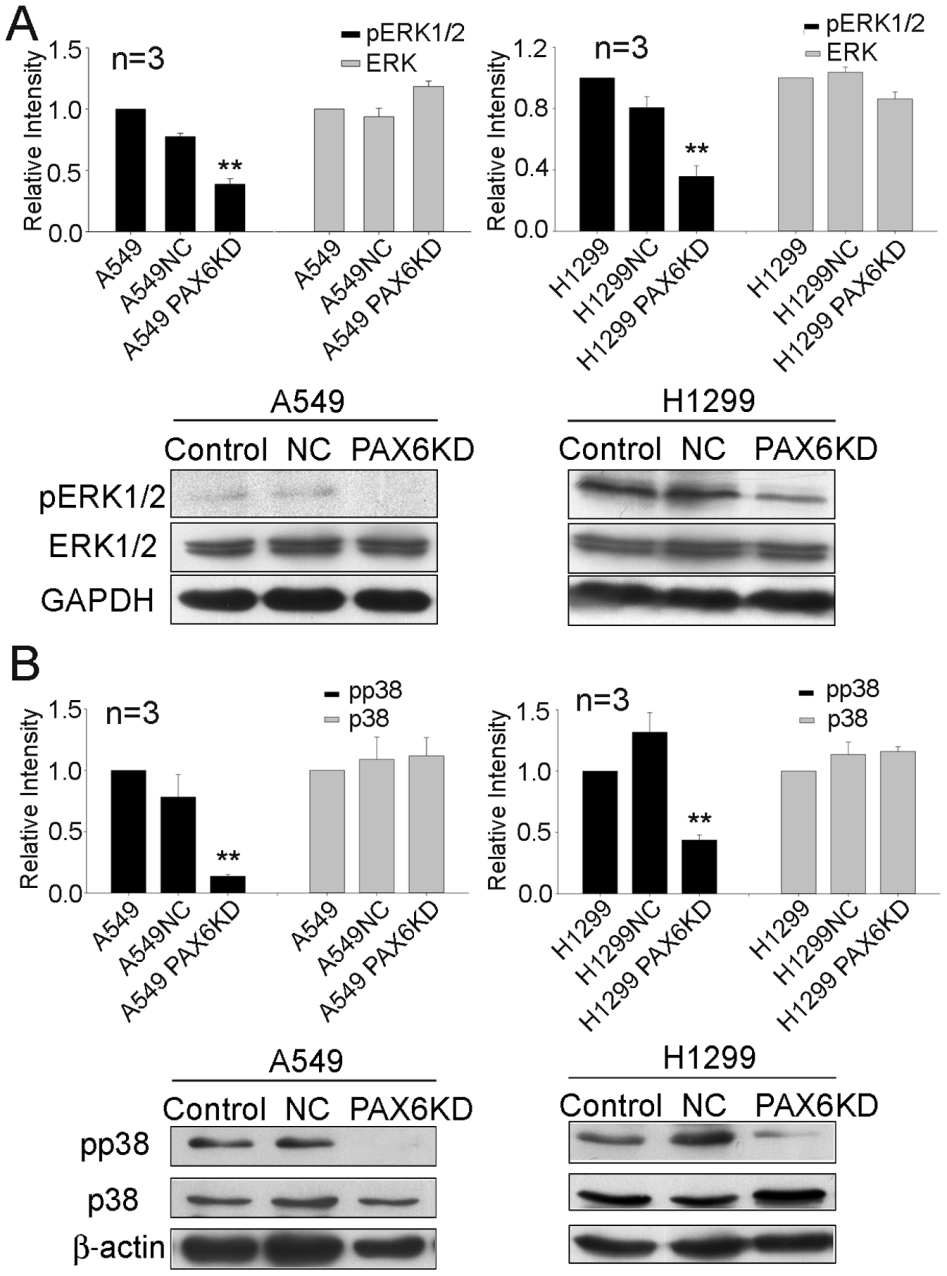
The phosphorylation levels of ERK1/2 and p38 were suppressed by the inhibiton of PAX6 expression. Western-blot analysis of A549, A549 NC, A549 PAX6 KD, H1299, H1299NC, H1299 PAX6 KD with antibodies to ERK1/2 (A), p38(B) and their phosphorylated forms were shown in the figure. GAPDH and β-actin was used as internal loading controls respectively. ERK1/2 and p38 levels were normalized by GAPDH and β-actin respectively. Data are expressed as mean ±SEM. All the experiments were repeated three times. *P <0.05, **P <0.01.

The MAPK (mitogen activated protein kinase) pathway has been implicated in the regulation of G1/S transitions and cell mitosis [19]. In our study, some central regulatory molecules of MAPK pathways were examined using western blot analysis. As shown in Figure 5, the phosphorylation levels of ERK1/2 and p38 were decreased both in A549 PAX6 KD and H1299 PAX6 KD cells. It indicated that the MAPK signal was weakened resulted from the RNAi interference of PAX6.

### PAX6 was highly expressed in lung cancer tissue

**Figure 6:**
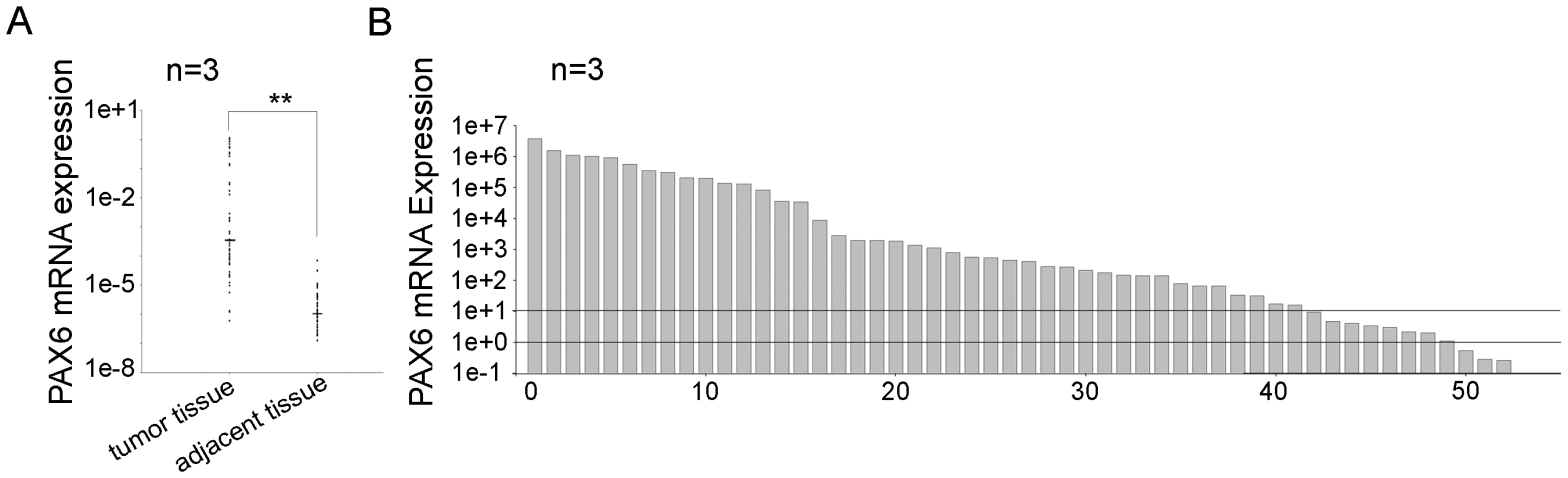
PAX6 mRNA was highly expressed in primary lung cancer tissues and lung cancer cell lines. A, Real-time PCR analysis of the PAX6 expression level in lung cancer tissues, as well as the matched adjacent tissues, from 52 patients. The PAX6 mRNA level was normalized by β-actin expression level. B, Each column represents the relative ratio of PAX6 mRNA in primary NSCLC versus adjacent lung tissue, and the line across the graph represents the value 1 and 10 respectively. All the experiments were repeated three times. **P <0.01.

Pax6 mRNA in lung cancer tissue, as well as matched adjacent tissue, was detected to confirm the role of PAX6 in lung cancer. The clinical characteristics of the 52 patients are listed in Table 1. As shown in Figure 6A, PAX6 mRNA was abundantly expressed in tumor tissue as compared to adjacent normal tissues. The expression of PAX6 represented by a cancer-to-adjacent nontumorous tissue ratio for each individual was indicated in Figure 5B. PAX6 expression in lung cancer tissue was higher than that in each matched adjacent normal tissue in all but three cases (Figure 6B). The statistic results were listed in table 2 and the ratio (tumor/adjacent tissue) of 65% patients (34 samples) exceeded 100. That is to say, in most cases, PAX6 was mainly expressed in lung cancer tissues.

**Table 2:** The relative ratio of PAX6 mRNA in primary NSCLC versus adjacent nontumorous lung tissue.

PAX6 mRNA level (Tumor/adjacent tissue)	0–1	1–100	100–10,000	10,000–10,000,000
Number of patients	3	15	19	15

## Discussion

In our study, the function of PAX6 in lung cancer cells was investigated. The growth ability of A549 and H1299 cells was declined when PAX6 expression was inhibited by specific PAX6 shRNA. We suggest that PAX6 promotes G1-S progression by activating the MAPK signal pathway. And PAX6 was highly expressed in lung cancer tissues and lung cancer cell lines.

The transcription factor PAX6 plays different roles in different tumors. It is frequently expressed in pancreatic cancer and retinoblastoma cells, implicating an oncogenic function, while PAX6 is recognized as a tumor suppressor in gliomas and prostate cancer [6], [10], [11], [20], [21].PAX6 expression is significantly reduced in glioblastomas and the expression level is correlated with longer patient survival [22]. PAX6 suppresses glioblastoma cell growth, anchorage-independent growth and glioma angiogenesis as well as invasiveness of glioblastoma cell, via inhibition of matrix metalloproteinase-2 (MMP2) expression and vascular endothelial growth factor A (VEGFA) expression [20], [23], [24]. In prostate cancer, PAX6 expression was lower in cancer tissues and cancer cell lines than normal epithelial cells [21]. Overexpression of PAX6 suppressed the proliferation and colony formation of prostate cancer cells [8].

But PAX6 plays an oncogenic role in pancreatic cancer and retinoblastoma [4], [22].In pancreatic adenocarcinoma and pancreatic cancer cell lines, down-regulation of PAX6 by specific siRNA leads to a decline in cell growth and cell apoptosis [12]. Methylation of PAX6-promoters is increased in early bladder cancer and methylated PAX6-promoters could be a represent biomarker for this disease [25]. And the suppression of PAX6 mRNA expression resulted in an inhibited growth and an increased apoptosis of cultured human retinoblastoma cells [26]. Our findings also reveal that PAX6 implicates an oncogenic function in lung cancer. In our results, PAX6 mRNA was highly expressed in both lung cancer tissues and lung cancer cell lines. A549 and H1299 cell growth was inhibited by specific PAX6 shRNA. Suppression of PAX6 expression led to decreased cell growth and colony formation, as well as anchorage-independent colony formation. But our findings indicate that cell apoptosis was not affected by the inhibition of PAX6 (data not shown). And cell migration was also not affected by the suppression of PAX6 mRNA (data not shown).

PAX6 is cancer-dependent and has different signaling pathways in different tumors [13], [20]–[24], [26]. In HeLa cells, PAX6 regulates cell-cycle progression by eliciting the expression of human RFPL1 (hRFPL1), which down-regulates cyclin B1 and Cdc2 expression and leads to the accumulation of cells in G2-M phase [27]. We also focused on the role of PAX6 in regulating cell cycle progression in lung cancer. In our cell cycle analysis, cyclin D1 was suppressed in A549 PAX6 KD and H1299 PAX6 KD cells. It indicated that PAX6 expression promoted cell cycle progression by transitioning cells from G1 to S phase. Consistent with these findings, cell cycle analysis showed a significant reduction of G0/G1 arrest and a significant induction of G2/M arrest in PAX6 overexpression human retinoblastoma cells [28].

Cyclin D1-CDK4 and cyclin D1-CDK6 complexes in the early to mid-G1 phase phosphorylate and inactivate pRB [29], [30]. Our findings in this study implicate that the pRB S780 phosphorylation level was weakened when PAX6 expression was inhibited. Thus, we demonstrated that PAX6 increased the expression of cyclin D1 and enhanced cell growth by promoting the G1-S transition. Mitogen-induced Ras signaling promotes transcription of the cyclin D1 gene and it depends on the MAPK signal pathway [31]. Our findings indicate that inhibition of PAX6 decreases the phosphorylation level of ERK1/2 and p38. These studies suggest that PAX6 regulates cell G1/S progression via MAPK signal pathway in lung cancer cells.

However, the regulatory mechanism of PAX6 in lung cancer is still unclear. A recent study showed that PAX6 promotes cell growth by activating the MET tyrosine kinase receptor gene in pancreatic carcinoma [13]. In lung cancer cells, ERK1/2 signal pathway is involved in the MET pathway [32]. Our finding indicated that ERK1/2 was activated by PAX6 expression. So that we suppose that PAX6 activates MAPK signaling and promotes cell cycle progression via MET gene transcription in lung cancer.

PAX6 is primarily expressed during embryogenesis; little or no PAX6 protein is detected in adult tissues [3]. As PAX6 is frequently expressed in tumors [9], we determined the PAX6 level in primary lung cancer tissues. The PAX6 expression level in matched adjacent tissues was measured as a control. Similar to pancreatic tumors, PAX6 expression was stronger in lung cancer tissues than in adjacent tissues. The cancer-to-adjacent nontumorous tissue ratio of PAX6 mRNA expression for each individual was calculated. Only 3 ratios were less than 1 and most of the ratios were much more than 100. All these findings demonstrated that PAX6 functioned as an oncogenic factor in lung cancer.

## Conclusions

In this study, we report that increased expression of PAX6 was noted in primary lung cancer tissues. PAX6 promoted cell growth by activating MAPK signalling and accelerating cell cycle progression. Moreover, PAX6 regulated G1-S progression by inducing cyclin D1 expression and pRB phosphorylation. Our data suggests that PAX6 is a new potential target in lung cancer.
